# “We are on the frontlines too”: A qualitative content analysis of US social workers' experiences during the COVID‐19 pandemic

**DOI:** 10.1111/hsc.13963

**Published:** 2022-08-06

**Authors:** Julie A. Cederbaum, Abigail M. Ross, Lisa de Saxe Zerden, Lilly Estenson, Jennifer Zelnick, Betty J. Ruth

**Affiliations:** ^1^ Suzanne Dworak‐Peck School of Social Work University of Southern California Los Angeles California USA; ^2^ Graduate School of Social Service Fordham University New York City New York USA; ^3^ School of Social Work University of North Carolina at Chapel Hill Chapel Hill North Carolina USA; ^4^ Leonard Davis School of Gerontology University of Southern California Los Angeles California USA; ^5^ Touro College Graduate School of Social Work New York City New York USA; ^6^ School of Social Work Boston University Boston Massachusetts USA

**Keywords:** COVID, essential workers, frontline workers, policy, social work

## Abstract

Social work has been a part of the essential workforce historically and throughout the COVID‐19 pandemic, yet lack recognition. This work explores the experiences and invisibility of social workers within the pandemic response. Data are drawn from a large cross‐sectional survey of US‐based social worker from June to August of 2020. A summative content analysis of responses to the question ‘What do you wish people knew about social work during the COVID‐19 pandemic’ was undertaken. Participants (*n* = 515) were majority white (72.1%) and female (90.8%). Seven coding categories were subsequently collapsed into three domains: (1) meeting basic needs, (2) well‐being (emotional distress and dual role) and (3) professional invisibility (workplace equals, physical safety, professional invisibility and organisational invisibility). Meeting social needs requires broad‐based policies that strengthen the health and social safety net. Social workers have and will continue to play a critical role in the response, and recovery from COVID‐19. Organisational and governmental policies must expand to increase the visibility and responsiveness to the needs of social care providers.

What is known about this topic?

- Social workers, along with other members of the essential workforce, have navigated unparalleled challenges at work in the context of COVID‐19.
- Lack of recognition for social workers has led to frustration and a call for visibility.
- Understanding and uplifting the role of social workers illustrates how organisations and policies can prioritise a skilled workforce equipped to address complex social and health conditions.

What this paper adds?

- COVID‐19 has spotlighted the importance of social care interventions and the necessary workforce needed.
- Social workers' lack of recognition during the COVID‐19 response reflects society's limited acknowledgement of social care needs and the individuals who serve our most vulnerable populations.
- Formal and informal recognition of social care workers is essential for retaining a skilled and necessary workforce.

## 1 INTRODUCTION

Globally, the COVID‐19 pandemic has highlighted challenges at the intersection of health, social needs and community. In addition to disruption of the ways in which people receive health and social care, the negative impacts of COVID‐19 on the well‐being of health and social care providers have been enormous (World Health Organization [WHO], 2021). Around the world, COVID‐19 has increased occupational risks associated with contracting or passing the virus, increased fatigue, exhaustion and symptoms of burnout, and in the most severe cases, led to suicide (WHO, 2021). Taking on new roles within one's organisation (including learning and implementing added protocols and standards) and workforce shortages has further exacerbated these conditions. This compounded strain has—and continues to have—a significant impact on frontline health and social care workers (WHO, 2021). In the United States (US), the country that has experienced more than 72 million COVID‐19 cases and accounts for nearly 16% of worldwide COVID‐19‐related deaths (John Hopkins University, 2022), the workforce has experienced unique challenges because of the structure of health and social safety nets. In the US, the social safety net consists of many different programs to meet various needs (e.g. health insurance, food support, housing) that are decentralised and administered in disjointed ways based on each States' policies and budgets, not through universal federal programming. Since each State sets the requirements of most safety‐net benefits, not every eligible individual receives the same amount of support. Likewise, without a national health insurance plan, healthcare coverage is inequitably distributed. In the US, the primary source of health insurance access is through employer‐based insurance programs (Karpman et al., 2020), followed by public programs for low‐income and/or vulnerable individuals, through means‐tested public insurance programs (i.e. Medicare, Medicaid, Children's Health Insurance Program) which vary by State. As communities shut down in the early phases of the COVID‐19 pandemic, unemployment in the US skyrocketed to nearly 15% in April 2021 causing not just economic hardship, but a loss of health insurance coverage to millions of workers (Fonstein & Woodbury, 2021).

COVID‐19 has shifted what society deems as essential work. In the US, essential work has been defined by the Centers for Disease Control and Prevention (2022) as a worker who is ‘essential to maintain critical infrastructure and continue critical services and functions’. Although often unrecognised, social work has been a part of the essential workforce historically and throughout the COVID‐19 pandemic (Guerrero et al., 2020; International Federation of Social Workers, 2022). This lack of recognition has led to frustration among social care practitioners, professional organisations and policy makers, leading to a call for visibility and recognition of social workers as part of the frontline pandemic response in the US (Abrams & Dettlaff, 2020) and abroad (Banks et al., 2020). In this paper, we explore US‐based social workers' responsibilities and recognition within the workplace and society in their role as essential workers during the COVID‐19 pandemic. Although this study focused on US‐based social workers specifically, findings have implications for social workers and other social care providers globally who also lack visibility (Paul et al., 2020) and have experienced high rates of overwhelm that leads to burnout (Banks et al., 2020; Downing et al., 2020; Miller & Reddin Cassar, 2021) during the COVID‐19 pandemic (and in its aftermath).

### 1.1 Social work and the COVID‐19 response

Frontline workers are essential workers at the highest risk for work‐related COVID‐19 exposure because their work duties are performed in‐person and with close proximity to the public (CDC, 2021; WHO, 2021). While many social workers were able to shift to telehealth during the early stages of the COVID‐19 pandemic (e.g. Cooper & Zerden, 2021; Lombardi et al., 2021; Ross et al., 2021), meeting human needs requires direct in‐person services in the domains of child protection, health, homelessness, substance use and aging/older adult services (Abrams & Dettlaff, 2020; Banks et al., 2020). In this capacity, social workers remain both frontline and essential. Social workers, along with other essential workers, have had to address clients increased emotional and health issues, along with their own, during a time of marked health, social and economic upheaval (Evanhoff et al., 2020; Shepherd & Newell, 2020). Because frontline social workers practice in environments that place them at higher risk for exposure to COVID‐19, threats to personal safety have remained a concern; fear of familial exposure has added to essential workers' burden and the gravity of navigating professional responsibilities with personal demands (Demartini et al., 2020). Together, these challenges have placed social care providers at increased risk of mental distress.

### 1.2 Lack of visibility and recognition of the social work profession

Social workers provide essential services to vulnerable populations, including during pandemics, yet are rarely recognised for their key roles in pandemic response (Paul et al., 2020). This is consistent with social work's historic invisibility and stature within health systems, and to a degree, within the broader society (Guerrero et al., 2020). This lack of visibility places social workers in a complicated position. While they often function as the ‘go to’ professionals for managing the mental and psychosocial needs of clients and patients (Forenza & Eckert, 2018) and participate on interprofessional teams (Zerden et al., 2018), workplace policies often fail to explicitly acknowledge social workers as frontline workers. During the pandemic, this had serious implications for in‐person social workers, who were often not included in personal protective equipment allocation (PPE; Redondo‐Sama et al., 2020) or eligible for hazard pay (Ross et al., 2021). Moreover, social workers were asked to expand the scope of practice within their roles. For instance, social workers provided vital emotional and practical support to their colleagues in the form of support groups, debriefing opportunities and warmlines (Viswanathan et al., 2020), while simultaneously providing services to patients.

In light of these inherent risks accompanied by expansion in role, social work leaders around the world undertook efforts to elevate social work's visibility during the COVID‐19 pandemic (e.g. UNICEF, 2021). In the US, social workers can have either a bachelor's degree (BSW) or a master's degree (MSW) to practice; depending on the State in which they reside, social work licensing exams might be needed to use the social worker title (Social Work Guide, 2020). There is no national licensure for social workers. Those who hold an MSW adhere to the clinical hours and testing requirements of the State they work in. All degree granting social work programs are overseen by an accrediting body (Council on Social Work Education, 2022), while oversight for licensure is overseen by each State's accrediting board (ex. California Board of Behavioural Sciences). Social work (MSW level) is recognised as the primary provider of social care in health settings (National Academies of Science, Engineering, and Medicine, 2019) and a key member of the health workforce (Ross & Zerden, 2020), yet there is evidence to suggest that roles and responsibilities of social workers are misunderstood (Ross et al., 2021). In health settings, the clarity of role responsibilities among interprofessional team members is essential to improving patient care (Bittner, 2018). We use Role Theory (Biddle, 1986) in this work to understand social workers' experiences of invisibility during the COVID‐19 pandemic. In this theory, one's role is posited to have behavioural associations; when there is role ambiguity, or lack of clarity in professional roles, this may precipitate role conflict and interfere with individual and organisational functioning. Within the workplace context role clarity can foster both individual and organisational well‐being, through reduced stress and improved well‐being (Hogg, 2000). As such, the purpose of this study was to understand what US‐based social workers wanted others to know about social work roles, responsibilities and practice during the COVID‐19 pandemic. Understanding the roles of social workers elevates more than the profession, it undergirds and illustrates how organisations and policies can prioritise a skilled workforce equipped to address complex social and health conditions during the ongoing COVID‐19 pandemic.

## 2 MATERIALS AND METHODS

Data for this qualitative content analysis were drawn from a cross‐sectional survey examining the experiences of US‐based social workers during the COVID‐19 pandemic (Ross et al., 2022). Data were collected between June and August of 2020 via an anonymous electronic survey that included both closed and open‐ended questions and took about 15–20 min to complete. Topics covered in the survey included practice changes, stress and burden and safety/access to PPE. The survey was anonymous and completed using a Qualtrics link (Qualtrics, 2017). Participation was voluntary and no compensation was provided. All procedures were approved by the Institutional Review Board at Fordham University and the National Association of Social Workers (a requirement for distribution through their listserve).

### 2.1 Recruitment

Snowball sampling was used to recruit practicing social workers in the US and associated territories. Recruitment of respondents occurred primarily through a collaboration with National Association of Social Workers National Office who distributed survey invitations to executive directors of State chapters with the request to disseminate the survey to membership listservs, social media outlets and message boards (Ross et al., 2021). The research team also distributed e‐mail invitations that included the survey link to 12 professional organisations (see Acknowledgments).

#### 2.1.1 Participants

Individuals were eligible to participate if they reported being a US‐based social worker practicing in any capacity. Due to the nature of the sampling strategy, it is not possible to report response rates. Of the 4083 initiated responses, 964 (24%) responses were excluded due to the following factors: the absence of demographic information (*n* = 917; 23%), international or duplicate IP address (*n* = 40, 1%), retiree status (*n* = 11; less than 1%) and spam responses (*n* = 7; less than 1%). Among the 3118 self‐identifying US‐based social workers who had complete survey data, 2012 (64.5%) responded to the open‐ended question, ‘What do you wish people knew about social work during the COVID‐19 pandemic?’ Of those, 515 respondents (25%) included some discussion of social workers being either frontline workers, essential workers, social care providers and/or meeting basic needs of vulnerable individuals.

### 2.2 Measures

#### 2.2.1 Demographic characteristics

Data were collected on a number of demographic variables including gender (male, female, non‐binary, gender not represented on this list or prefer not to disclose), race (American Indian/Alaska Native [AIAN], Asian American/Pacific Islander [AAPI], Black/African American [Black] and white)/ethnicity (Hispanic/Latinx or non‐ Hispanic/Latinx), age (in years) and years in practice (in years).

#### 2.2.2 Workplace characteristics

Respondents identified their current role, selecting one of the following designations: direct care provider, student, manager/program manager, administrator/director, policy practitioner, researcher, academic or other. Practice setting was a multiple‐choice question initially spanning 20 areas of practice (academic/research, schools, early childhood (0–5), hospital, community health, community mental health, Department of Public Health (DPH), aging/older adult, child welfare, housing/homelessness, substance use treatment, domestic violence, criminal justice, public assistance, employment and training, family services, immigration services, disability services, other government agency and international organisation). Due to small cell sizes in some response categories, these were collapsed into the following: academic/research, school, hospital, community health, community mental health, substance use, children/youth/family services, aging/older adults/disability services, international, DPH, non‐DPH government agency, private practice and other.

#### 2.2.3 Geographic region

Participants were asked to report the US county and State where they worked. State‐level information was recoded to reflect membership in one of the 10 Health and Human Services (HHS) designated geographic regions: Region 1 (Boston), Region 2 (New York), Region 3 (Philadelphia), Region 4 (Atlanta), Region 5 (Chicago), Region 6 (Dallas), Region 7 (Kansas), Region 8 (Denver), Region 9 (San Francisco) and Region 10 (Seattle). Because it has the largest number of respondents, Region 9 was used as the referent group.

#### 2.2.4 Qualitative analyses

Based on the nature of the open‐ended questions, which were included as part of a larger quantitative study, and the size of the sample, we used a qualitative descriptive approach (Sandelowski, 2000) with a summative content analysis to analyse data (Hsieh & Shannon, 2005). A summative content analysis approach begins with the identification and counting of certain words or text phrases with the goal of understanding, but not interfering with the meaning, to explore how individuals describe an experience (Hsieh & Shannon, 2005). A form of qualitative descriptive methods, this type of approach allows for the presentation of experiences in everyday language, with words serving as the instrument for communication, not an interpretive structure (Sandelowski, 2000). This method allows questions to be answered very directly, making findings particularly salient for practice and policy changes. Two authors were responsible for data analysis. The first coder (first author), a trained clinical social worker and social work academic, has extensive training in qualitative methods and has led US federally funded qualitative research projects. The second coder (fourth author) is a masters level social worker and current doctoral student who was trained for these analyses by the first author. All data were entered into an excel file with several tabs (one with all data, and one tab for each coder). Excel was used because of the volume of respondents and the brevity of responses.

The first author began by reviewing all unique text responses (*N* = 2012) using an emergent coding approach (Stemler, 2000) to identify sensitising concepts (Bradley et al., 2007), yielding a total of 515 text samples. Next, coders (first and fourth author) reviewed all included text samples using open coding. Coders met regularly to discuss categories that emerged from sensitising concepts and subsequently finalised the codebook. Upon completing this phase of analysis, 10 text samples that did not clearly fit identified codes were dropped from the sample, resulting in a final sample size of 505 text responses. Both coders then completed a second review (coding) of all text responses using the finalised codebook; a 0 (not in the text sample) or a 1 (in the text sample) was given to each response in at least one of seven categories (1) meeting basic needs; (2) workplace equals; (3) physical safety; (4) emotional distress; (5) dual role; (5) professional recognition, (6) organisation recognition; and (7) policy recognition. Coders met weekly to discuss coding challenges and/or questions. Differences were discussed and choices made on how to proceed using audit trails to note decision‐making. Inter‐coder reliability (ICR) was run using SPSS v.27 (IBM Corp, 2019) on each distinct category (meeting basic needs; workplace equals; physical safety; emotional distress; dual role; professional recognition; organisation recognition; and policy recognition) to test coder agreement (O'Connor & Joffe, 2020). ICRs compared the two coders on their designation of a 0 or 1 for a given participant in a given category (assessed using Cohen's kappa coefficient); agreement ranged from 0.80 to 0.87. A coefficient of 0.80 was used as the minimal threshold because it is the minimum value for a strong level of agreement (McHugh, 2012). Reviewers had a final meeting to discuss the collapsing of codes into themes, grouping like concepts.

#### 2.2.5 Quantitative analyses

Univariate statistics were used to describe the study sample. Upon completion of coding, all data were entered into SPSS v.27 (IBM Corp, 2019) for analyses. Content analysis with representative quotes are presented below. Next, chi‐square analyses were undertaken to understand the relationships between sample characteristics and study codes (workplace equals, physical safety, emotional distress, dual roles, professional invisibility and organisational/policy invisibility).

## 3 RESULTS

Sample demographics are presented in Table 1. Participants identified predominantly as white (72.1%) and female (90.8%). Mean number of years as a social worker was 16.9 years. There was representation from all regions of the US (as defined by Health and Human Services, 2021) with the largest number of participants coming from Regions 9 (23.8%; San Francisco), 2 (16.2%; New York) and 4 (13.3%; Atlanta). Twenty workplace settings were identified, with the largest being hospital or community health settings (31.9%), followed by community mental health (14.3%), and private practice (10.2%); 79.4% of all participants reported serving in a direct practice role. There were no statistically significant differences in any demographic characteristics except in Region 9 (San Francisco), where there were a significantly higher number of participants who self‐identified as Black, (AAPI), Native American and/or Latinx (48.3%; *p* < 0.001).

**TABLE 1:** Sample demographics and workplace characteristics (*N* = 505)

Demographics and workplace characteristics	M	SD
Number of years in practice	16.9	11.9

	*N*	%
Race/ethnicity		
American Indian/Alaska Native	4	0.8
Asian American/Pacific Islander	22	4.4
Black	49	9.8
Latinx	44	8.8
Multiracial	20	4
White	360	72.1
Gender		
Female	454	90.8
Male	36	7.1
Non‐Binary/prefer not to disclose	10	2.0
Work setting		
Academic/research	19	3.8
School	23	4.6
Hospital	108	21.5
Community health	52	10.4
Dept of PH/other gov	49	9.8
Private practice	51	10.2
Children, youth and family services	37	7.4
Community mental health	72	14.3
Substance abuse	19	3.8
Aging/older adult/ disability	35	7.0
Other	37	7.4
Region		
R1 (Boston)	56	11.1
R2 (New York)	83	16.4
R3 (Washington, D.C.)	37	7.3
R4 (Atlanta)	69	13.7
R5 (Chicago)	57	11.3
R6 (Dallas)	27	5.3
R7 (Kansas City)	20	4.0
R8 (Denver)	10	2.0
R9 (San Francisco)	121	24.0
R10 (Seattle)	22	4.4
Role		
Direct service provider	396	79.4
Manager	52	10.4
Administrator/director	37	7.4
Academic	14	2.8

Data identified using the sensitising concepts were coded into one of the seven categories. Coding of an individual's response was not restricted to one code. The per cent reported shows the frequency of sensitising concepts within the data: (1) workplace equals in provision of services (16.7%); (2) physical safety concerns (16.9%); (3) experiencing emotional distress (10.3%), (4) responding to basic needs (31.7%), (5) playing a dual role (4.4%); (6) professional invisibility (55.2%) and (7) organisation and policy invisibility/inclusion (20.4%). There were several significant differences in responses to sensitising concepts by demographic characteristics. Individuals who identified as non‐binary/other were significantly more likely to discuss organisation/policy invisibility/inclusion (*p* < 0.011). In addition, there were regional differences in reporting of professional invisibility, with Region 7 (Kansas City) most likely to discuss lack of social work professional invisibility (*p* < 0.009). Workplace setting was associated with the discussion of emotional distress and meeting basic needs of clients with those in substance use treatment settings being more likely to discuss emotional distress (*p* < 0.002) and those working in children, youth and family settings more likely to discuss meeting basic needs (*p* < 0.031).

Open‐ended responses from the aforementioned seven sensitising concept categories were subsequently collapsed into three domains: (1) meeting basic needs, (2) well‐being (emotional distress and dual role) and (3) professional invisibility (workplace equals, physical safety, professional invisibility and organisational invisibility). Domains are described below and representative quotes can be found in Table 2. To reflect the experiences of social work respondents by their nuanced identities, parentheses that follow quotations denote the respondent's race/ethnicity (AAPI, Black, Latinx, white), Years in practice (number) and Region (R1–R9).

**TABLE 2:** Themes and illustrative quotations from the perspective of participants

Themes	Subthemes	Illustrative quotes
1. Basic needs	1.1 Increased vulnerability	*Social workers have continued the mission, both paid and voluntary, throughout this period. It is our calling and our duty to do so*. (White, child welfare, 33, R4) *People need to be linked to resources more than ever as jobs, housing and food is an ever‐increasing issue, especially in already disadvantage areas*. (Black, community health, 19, R4)
	1.2 Increased need for services	*Just because COVID‐19 occurred does not mean the need for our services has disappeared, if anything it has greatly expanded, and again, has highlighted the lack of capacity, funding, and resources many organisations have to actually live out their missions*. (White, housing/homelessness, 7, R5) *When the spate of mental health related issues takes hold as a result of the pandemic and disproportionate effect on communities of colour and the working class in general, social workers will be the frontline of defence against the scourge of joblessness, suicide, PTSD, homelessness, and poverty*. (White, aging/older adult, 6, R2)
2. Well‐being	2.1. Emotional distress	*The demand for increased services continues to pile on without more time or support to meet the drastically increased workload. Our mental health continues to decline and many of us are struggling with depression and anxiety, with no support. I am emotionally drained and exhausted to the core of my being*. (White, community mental health, 10, R8) *I was redeployed from outpatient medicine to inpatient medicine and worked on a floor that was a hybrid of a non‐COVID recovery room and ICU. I did an average of 5 family video visits a day for 26 days with a total of about 130 video visits during my redeployment. I did all of these visits in‐person, at bedside, in full PPE. I was discharge planning, crisis counselling, facilitating bereavement sessions, and it was my job to tell families where their loved ones' bodies went and how to arrange death services without families being physically present! I have really been through a lot*. (Asian/Pacific Islander, hospital, 1, R2) *I believe it is important for others to know [that] strong social workers have suffered emotional and mental stress during the time while trying to be strong for everyone else*. (White, community health, 16, R7)
	2.2 Dual role	*In my experience during this crisis social workers have worked tirelessly to come up with creative ways to provide psychosocial, emotional, and resource support remotely…while also coming together professionally to support self‐care and combat burnout*. (White, aging/older adult, 8, R9) *that social workers are also at the ‘frontlines’ and are often supporting their medical clinical colleagues in counsellor roles*. (Asian/Pacific Islander, hospital, 6, R10) *For me, as a social worker in a hospital setting, I find myself being a social worker for not only my patients, but for my fellow RN, MD and NP colleagues*. (Latinx, hospital, 2, R9) *It's very emotionally draining. We are social workers in our work life, but then after hours is often assisting friends and family members in navigating resources, being a person they feel comfortable talking to, etc. there is not really any sort of break*. (unreported race, community health, 6, R5)
3. Professional invisibility	3.1 Workplace equals	*We are here, every day, in the hospital putting ourselves at risk alongside the nurses and the doctors*. (White, hospital, 9, R3) *The role of social workers is typically overlooked. They are often integral parts of healthcare teams along with doctors and nurses in healthcare settings*. (Asian/Pacific Islander, private practice, 10, R9) *I am in health care and as at much risk as the nurses—actually more due to the length of time I have with each patient—processing emotions takes face to face time*. (White, international, 28 years, R1)
	3.2 Physical safety	*The risks we take are unlimited. I contracted COVID‐19 from clients who consequently passed away*. (White, community mental health, 30, R2) *Social workers are also at risk of exposure to COVID and are making sacrifices to serve*. (Asian/Pacific Islander, hospital, 6, R10) *Social workers continue to provide services, ensure safety of children, and do so without adequate supplies and/or direction in some circumstances. Social workers rise up to meet the challenges in the community even when it means putting those in their own homes at risk*. (White, child welfare, 10, R9)
	3.3 Professional visibility	*WE ARE ESSENTIAL. We are the least funded yet we are usually our clients' first phone call. We are still doing home visits and counselling appointments but we are not being provided with PPE, hazard pay, or overtime. The community does not know we are out there, but we are the first line of defence for so many vulnerable groups*. (White, community mental health, 8, R2) *Social Workers have been furloughed or have lost their jobs at hospitals just like many other medical professionals. Social Workers do not always qualify to get hazard pay. Social Workers often make significantly less money than their medical peers even though they may have more experience and more education*. (White, Department of Public Health, 16, R4) *We need to advocate for hazard pay and inclusion in any possible loan forgiveness programs for medical professionals /front line workers*. (White, community mental health, 6, R1)
	3.4 Society visibility	*Social workers are not publicly recognised as essential workers. This is demeaning to the value social worker place on their work toward a better more equal life for all. I am disheartened that social workers have not been thus recognised*. (White, aging/older adult, 6, R2) *We do the hard work, but no one sees us, understand or cares of the sacrifices and dangers that we faced everyday*. (Black, community mental health, 11, R2) *It seems like people forget that social workers are also frontline staff in hospitals, community mental health, hospice, schools, child welfare and so on. I do not hear any talk about social workers when I watch corporate or non‐corporate news but I know that they are suffering and vulnerable along with those that are acknowledged EMTs, doctors, nurses, cops. People should know that we are the unsung heroes out there working in diverse practice settings to provide essential services*. (Black, hospital, 5, R8) *We are frontline workers and doing our best to advocate for and provide services in the face of being deemed non‐essential and facing fears about future funding. We are the leaders in creating societal change for the better in response to COVID*. (Multiracial, disability services, 1, R9)

### 3.1 Basic needs

Close to one‐fifth (*n* = 100; 19.8%) of social workers discussed their role as meeting the basic needs of clients, which predated the pandemic, but which was amplified by COVID‐19‐related shutdowns. Meeting basic needs included attending to issues like housing needs, and income and food insecurity. Respondents also described that the shutdowns left vulnerable populations at increased risk for negative outcomes (a significant concern given baseline vulnerabilities). Social workers predicted that beyond those already vulnerable, it was likely that increased mental health needs would arise among new groups (see Table 2 for sample quotes).

Social workers also shared how COVID‐19 would likely exacerbate challenges for already vulnerable populations, lending to an even greater need for social care provision. As expressed by this social worker. Overall, individuals perceived people as needing social workers more than ever before. This response is grounded in the values of social work practice and is overwhelming seen by social workers as their role in the response to social need. Respondents also observed that while medical providers might see a decline in their workload as COVID‐19 stabilises, the expected mental health‐related challenges resulting from pandemic‐related isolation, loss and economic insecurity would land squarely on the shoulders of social workers. Organisational capacity, funding and resources were all noted as potential barriers to meeting the population's increasing basic needs.

### 3.2 Well‐being

#### 3.2.1 Emotional distress

Just over 10% (*n* = 52) of respondents discussed the emotional toll of working during the COVID‐19 pandemic. Emotional distress was related to individuals' own fears for their safety, particularly among social workers who were required to work in‐person. Respondents described distress related to placing their own families at risk, having an increased workload with no commensurate increase in resources, noticing increased client need—which they felt helpless to address—and feeling the emotional burden of attending to the level of distress being experienced by their clients. Other participants described feelings of overwhelm and fatigue. Ultimately, having to be strong for those around you while managing one's own worries lent to overwhelm and fatigue.

Social workers, along with others, had their own fears related to personal protection from COVID‐19; this was particularly true for social workers who did not have the option to work remotely. For those working remotely, the struggle included keeping work‐life and home‐life separate. Increased demand for both in‐person and remote services (due to the impact of the COVID‐19 lockdown on the mental and physical health of our communities) further taxed social workers whose well‐being declined because of this added stress. These significant changes to the way in which social workers carried out their jobs (and the need to quickly modify strategies) likely contributed to role strain which further influenced well‐being.

#### 3.2.2 Dual role

Playing a dual role (*n* = 22; 4.4%) also emerged as a salient theme challenging social workers' well‐being. Most frequently described by social workers employed in health settings, dual role was described as the provision of mental health to clients, in addition to provision of formal and informal mental health support to colleagues. Respondents also noted that these services were often designed to address colleagues' own sense of overwhelm or emotional process challenges associated with increased patient deaths from COVID‐19. Last, social workers also described a second dual role of serving both their client population and also providing guidance and support to family and friends. These dual roles led to feelings of exhaustion, challenges to engagement in self‐care and overwhelming feeling of needing to attend to the emotional needs of others. Having to take on multiple roles may have contributed to role ambiguity and increased strain on social workers, impacting their overall well‐being.

### 3.3 Professional invisibility

Issues related to the visibility of social work arose in a number of ways. More than half of the sample shared lack of perceived professional visibility in the workplace by colleagues, organisational leadership and in organisational policies (*n* = 278; 55%). Feelings of invisibility were particularly salient for hospital social workers who described working alongside physicians and nurses as part of COVID‐19 treatment teams. These social workers described that not being treated as workplace equals made them feel undervalued by colleagues. This perception was also described at the organisational level. Even when deployed to work in person, social workers described being required to work in person without PPE to keep them safe. Social workers across multiple settings described increased caseloads with no overtime or hazard pay, although interprofessional colleagues in nursing and medicine were frequently offered these benefits.

Societal invisibility was also a predominant theme. Of the 505 participants, 209 (42.4%) used the term ‘essential’ when describing what they wanted people to know about social work practice during the COVID‐19 pandemic. Another 51 wanted wider social recognition of social workers as frontline workers. As such, over 50% of the sample described the desire for society to recognise social workers as frontline and/or essential workers. Social workers also described feeling disheartened when left out of campaigns to support ‘healthcare heroes’, undervalued when not included in nightly cheers, excluded when not highlighted in media (along with other non‐medical providers such as firefighters and police), and generally undervalued and unappreciated for their labour during the COVID‐19 pandemic. Social workers further perceived the profession was being left out of conversations my elected officials related to provision of PPE and hazard pay. The following quotes highlight the feelings of invisibility shared by social workers.

Overall, it was clear that social workers both wanted and needed to be more visible. In their roles, social workers were part of interprofessional teams serving our most vulnerable populations, but because of role ambiguity or lack of role promotion, these efforts were experienced as unrecognised, and by default unappreciated by society at large. Role visibility and acknowledgement was noted as something social workers desired from colleagues, their organisations and society at large. This lack of recognition created frustration and left social workers disheartened (potential precursors to role dissatisfaction).

## 4 DISCUSSION

This is the first study drawn from a national US‐based sample of social workers practicing across various geographic areas and settings; it examined experiences of social workers practicing during the early phases of the COVID‐19 pandemic with a particular focus on frontline and/or essential status. To help understand the experiences of social workers during the COVID‐19 pandemic, this study examined the experiences of social work providers at the individual, organisational and national/policy levels (as has been suggested by prior work on Role Theory in the context of social work practice; Thompson & Greene, 2017). Role theory provided a framework for our findings related to social workers' commitment to their roles (meeting the needs of vulnerable populations), and challenges to well‐being and visibility (role ambiguity) as frontline workers. Our findings expand on prior studies of experiences of social workers during the COVID‐19 pandemic that have primarily focused on practice modifications undertaken at specific organisations or within a circumscribed geographic region or population (e.g. Cooper & Zerden, 2021; Cornell et al., 2021; Rubin & Rassman, 2021). While a few studies have investigated experiences of social workers and the impact of the COVID‐19 on social workers' well‐being (e.g. Miller & Reddin Cassar, 2021; Ross et al., 2021), these have been restricted to samples of social workers employed in health and hospital settings. Moreover, although calls for social work to be recognised as an essential workforce have proliferated in response to pandemic‐related needs of populations served both nationally and internationally (e.g. Gewirtz, 2020, UNICEF, 2021; Videmšek & Fox, 2021), to our knowledge, studies (in the US or other countries) have not thoroughly explored the ways in which the lack of acknowledgement of social work as essential and/or frontline has been experienced by social workers themselves.

The most noteworthy finding from this study is that over half of the final sample (55%) expressed a desire for both formal and informal recognition of social work are essential and, in many cases, part of the frontline workforce. The absence of recognition from workplaces and the general public took a psychological toll on social workers. Invisibility of the profession during the COVID‐19 pandemic has been reported by social workers in other countries as well, including India, Slovenia and Sri Lanka, where professional integration in responding to the global pandemic varied, but in all cases were needed and not recognised (Dominelli & Harrikari, 2020). Inadequate recognition and resources, partly related to the lack of a professional regulatory body, were also noted as a challenge for social care providers in Africa (Kodom, 2022). Lack of visibility has also manifested in inequitable treatment such as lack of hazard pay/compensation for overtime shifts and lack of PPE, most notably among those practicing outside of traditional healthcare settings. The implications of this are crucial. Social workers who experience higher levels of organisational support report lower levels of workplace stress (Senreich et al., 2020) and burnout (Hamama, 2012). In the era of COVID‐19, a sense of belonging and feeling valued has been identified as a salient protective factor against both stress and burnout (Sangal et al., 2020). Within the social work workforce specifically, qualitative research conducted with hospital‐based paediatric frontline social workers showed that acknowledgment and recognition of social work as core members of interprofessional teams fostered a sense of belonging, pride and psychological safety (Ross et al., 2021). These factors foster workplace well‐being, role satisfaction and quality job performance (Waller, 2020). Taken together, the finding suggests that recognition of social work as frontline/essential workers could function as a buffer against workplace stress exacerbated by the COVID‐19 pandemic, and in turn, protect against burnout and other adverse outcomes.

The second most prevalent theme was the role of social workers in response to increasing basic needs in the population. While this finding is not surprising given exponential growth in unmet basic needs during COVID‐19 (Suh et al., 2021), the settings in which this was most evident speaks to the vulnerabilities of those who were already at risk before the pandemic. For example, a significantly greater proportion of social workers employed in substance use treatment settings and those serving children and families reported more intense focus on meeting basic needs. These findings align with those of the Urban Institute's Health Reform Monitoring Survey, which identified substantial challenges related to food insecurity, housing and other basic needs (Karpman et al., 2020).

Finally, a substantial number of respondents indicated concerns about physical safety and associated emotional distress, phenomena that do not appear to be unique to US‐based social workers (e.g. Videmšek & Fox, 2021). In fact, studies from around the globe have noted the impact of the COVID‐19 pandemic on the well‐being of social care providers (UNICEF, 2020). Concerns related to personal health and safety were found in studies of social care providers in Canada (Ashcroft et al., 2022), Cambodia (Henley et al., 2021) and Finland (Saraniemi et al., 2022). Many of these same studies found that the COVID‐19 pandemic placed significant strain on emotional well‐being of social care providers. In a meta‐analysis, Li et al. (2021) found that more than 1/5th of care providers endorsed symptoms of anxiety, depression and/or PTSD. Impacts on psychological health and well‐being were also reported by social care workers in the United Kingdom (McFadden et al., 2021), and Ecuador (Ruiz‐Frutos et al., 2022). It is possible that the higher prevalence of emotional distress reported among social workers employed in substance use treatment settings may be rooted in fears about job security. Decreased revenue due to lower client censuses, staff furlough and layoffs, were all identified as concerns (Pagano et al., 2021). Changes to patients' insurance, treatment adherence and access to services (Ornell et al., 2020) may have further compounded social workers' emotional distress given limited in‐person service access at a time of increased substance use treatment needs among their clients. Likewise, the rate of US opioid overdose deaths worsened during COVID‐19 (Kuehn, 2021); it is plausible that social workers in substance use treatment settings were aware of the ramifications the pandemic would have on the clients they serve, increasing distress because of limitations placed on their role/access to those in need. Protecting against this distress can include the implementation of burnout prevention interventions to better support workers' stress and concerns based on practice settings and clients' needs. Additionally, another way to buffer this emotional distress is to elevate social workers' roles and promote their service and contributions during the pandemic. For example, a study in Spain found that building reliable webinars about social work services during their country's lockdown was a promising way to promote their work, counter misinformation and raise the public's awareness of social workers experiences during COVID‐19 (López Peláez et al., 2020).

### 4.1 Limitations

Although our findings offer important insights about experiences of US‐based social workers practicing during the COVID‐19 pandemic, there are several limitations. Most notably, we are aware that the data collected is from one country and does not allow for cross‐national comparisons, something we hope can occur in future research. Our sample was targeted for social workers in the US and is not generalizable to other countries where the structure and financing of care differs. Second, it is not possible to determine a response rate based on our sampling methods. Relatedly, there could have been response bias due to recruitment via professional organisation affiliations. Third, data presented here is cross‐sectional in nature and as such, do not account for COVID‐19‐related variations in new variants or regional outbreaks that may vary over time, or variability based on local or state mandates such as (physical) distancing and other pandemic mitigation measures (e.g. mask wearing). Lastly, our survey did not include a specific question about perspectives of frontline/essential work. While we did not ask a specific question, the fact that so many introduced this theme speaks to the need to recognise social workers as essential and frontline.

## 5 CONCLUSION

Perhaps more than ever before, COVID‐19 has spotlighted the importance of social care interventions and the necessary workforce to implement social and public health measures in the time of a global pandemic. Recently, Public Health 3.0 has been used to re‐conceptualise public health infrastructure that includes preventative approaches to address social determinants, risk and needs, by collaborating with community resources and assets (DeSalvo & Wang, 2017). COVID‐19 has forced the US to re‐assess who is defined as a frontline worker and why these workers are essential to the nation's social, economic and public health infrastructure (Blau et al., 2020). Inclusion of social work within this designation is necessary not only because it impacts the sustainability of an essential workforce, but without them, social needs worsen, especially in times of crisis like we find ourselves in now.

Perhaps not surprisingly, lack of recognition of social work during COVID‐19 reflect society's lack of recognition of social care needs, which in turn, adversely impacts public health's ability to address upstream issues related to health and well‐being emerging from COVID‐19. Amplifying how social workers contribute to frontline essential care will help to focus attention on unmet population needs and widen the lens of pandemic recovery. However, this will require both immediate and long‐term efforts and advocacy is needed in the form of change to policies including workplace safety measures (such as distribution of PPE), additional hazard pay and acknowledgment that mental health and social care are crucial to societal well‐being and that the workforce providing these services is ‘essential’ (Pollack et al., 2020). Long‐term efforts include increased re‐investment in educational pipelines to expand the public health social work workforce to be prepared and ready to respond to this crisis, and those to come. Actualizing Public Health 3.0 requires broad‐based policies that strengthen the health and social safety net. However, this work is not possible without acknowledging and supporting the critical role social workers play in society's response, and recovery, from COVID‐19. Social work's longstanding role in health and public health needs elevating and this, in turn, will help meet the social care needs of our communities.

## CONFLICT OF INTEREST

The authors have no conflict or financial interest to disclose.

## Data Availability

The data that support the findings of this study are available from the corresponding author upon reasonable request.
